# Effect of Cognitive Disability and Ambulation Status on Functioning in Moderate-to-Advanced Parkinson Disease

**DOI:** 10.3389/fneur.2019.01360

**Published:** 2020-01-09

**Authors:** Chen Yu Wang, Lung Chan, Dean Wu, Wen-Chou Chi, Chia-Feng Yen, Hua-Fang Liao, Chien Tai Hong, Tsan-Hon Liou

**Affiliations:** ^1^Department of Neurology, Shuang Ho Hospital, Taipei Medical University, New Taipei City, Taiwan; ^2^Department of Neurology, School of Medicine, College of Medicine, Taipei Medical University, Taipei, Taiwan; ^3^Taiwan Society of International Classification of Functioning, Disability and Health, TSICF, New Taipei City, Taiwan; ^4^Department of Occupational Therapy, Chung Shan Medical University, Taichung, Taiwan; ^5^Department of Public Health, Tzu Chi University, Hualien City, Taiwan; ^6^School and Graduate Institute of Physical Therapy, College of Medicine, National Taiwan University, Taipei, Taiwan; ^7^Department of Physical Medicine and Rehabilitation, Shuang Ho Hospital, Taipei Medical University, New Taipei City, Taiwan; ^8^Department of Physical Medicine and Rehabilitation, School of Medicine, College of Medicine, Taipei Medical University, Taipei, Taiwan; ^9^Graduate Institute of Injury Prevention and Control, College of Public Health, Taipei Medical University, Taipei, Taiwan

**Keywords:** Parkinson's disease, disability, cognition, ambulation, WHODAS 2.0

## Abstract

**Background:** As the disease progresses to moderate to advanced stages, people with Parkinson's disease (PwP) are likely to have various degrees of disability due to the motor and non-motor symptoms, such as ambulatory difficulty and cognitive impairment. The objective of this study was to investigate the impact of cognition and ambulation status on the functioning and disability of PwP using the World Health Orgnaization Disability Assessment Schedule 2.0 (WHODAS 2.0).

**Materials and Methods:** A group of 10,581 PwP with Hoehn and Yahr Staging 3 and above were collected from a database of disability evaluation and functional assessment using the Taiwan Data Bank of Persons with Disability between July 2012 and October 2018. WHODAS 2.0 was administered and all PwP were grouped based on their ambulatory status, which was assessed by 3-m back and forth walk and cognitive ability, assessed by WHODAS 2.0 first domain with cut-off level at 58.

**Results:** Non-ambulation and cognitive disability contributed independently to disability in all aspects of WHODAS 2.0 survey, including self-care, getting along with others, performing life activities and participation in society. Compared to ambulation status, cognitive disability had a greater negative impact on functioning in all aspects.

**Conclusion:** Cognitive disability was associated with greater disability in moderate to advanced PwP than non-ambulatory status. The results of this study may indicate that cognition preservation is essential to ameliorate functional impairment and disability in moderate to advanced PwP.

## Introduction

Parkinson's disease (PD) is the second most common neurodegenerative disease comprising of motor and non-motor features due to dopaminergic and non-dopaminergic deficiencies (1). In industrialized countries, the prevalence of PD is around 0.3% in the general population and 1% in those older than 60 (2). In 2016, 6.1 million individuals were affected by PD, causing 3.2 million Disability-adjusted-life-years. Global burden of this condition is expected to increase as a result of longer life expectancies, longer disease duration, and environmental factors (3).

Disability is defined as “the state of decreased functioning associated with disease, disorder, injury, or other health conditions, which in the context of one's environment is experienced as an impairment, activity limitation, or participation restriction”, hence it is fundamental to use a comprehensive assessment tool to examine all aspects that may hinder a person's ability to carry out normal daily activities, whether it may be instrumental activities of daily living, social participation, or environmental contributions (4). The WHO Disability Assessment Schedule 2.0(WHODAS 2.0) is a generic assessment tool developed in accordance to the conceptual framework of the International Classification of Functioning, Disability, and Health and is used to produce standardized disability levels and profiles applicable across different populations and health conditions from various countries including Taiwan with good reliability and validity (5–8). WHODAS 2.0 encompasses six domains of life: cognition (understanding and communicating), mobility (moving and getting around), self-care (hygiene, dressing, eating and staying alone), getting along (interacting with other people), life activities (domestic responsibilities, leisure, work, and school), and participation (joining in community activities). In Taiwan, The Functioning Disability Evaluation Scale Adult Version (FUNDES-Adult) was modified and translated from the WHODAS 2.0 with some minor modifications made to account for the Chinese culture. Domain 7 (environmental attributes) and domain 8 (motor action, capability and capacity scores) were added in order to increase comprehensiveness and to account for perceived environmental barriers (5, 6, 8, 9).

Regarding moderate and advanced PD, disability is traditionally thought to be associated with the core motor features of tremor, rigidity, bradykinesia, and postural instability; out of these features, postural instability is found to be most common in this population and a strong prognostic factor of determining progression to disability (10, 11). However, non-motor aspects of PD can also profoundly impact a person's level of disability, although the extent of their contribution can be oftentimes underestimated or even overlooked (12). Amongst the non-motor symptoms, dementia presents insidiously over the disease course, occurring in up to 40% of people with PD (PwP), which is six times higher than aged matched controls (13, 14). In The Sydney multicenter study of PD, the cumulative prevalence of PD dementia (PDD) is 83% over 20 years and the prevalence increases as age advances (15, 16). Both patients and caregivers frequently report cognitive decline as one of their greatest concerns and a major unmet need despite increasing recognition of PD as much more than a motor disorder (17). It has also been proposed that there are inter-relationships between motor function and cognition based on observations functional mobility is significantly correlated with cognitive impairment and that those with cognitive impairment demonstrate poorer motor function compared to matched PD patients without cognitive impairment (18, 19). Others have found faster rate of cognitive decline in those with postural instability (20). In addition, studies that looked at dual tasking found that even when they are treated optimally, PwP showed deterioration in gait parameters and that the degree of deterioration was correlated with baseline cognitive and mobility status (21). As one of the leading sources of disability globally, it is of utmost importance to understand the association between the major symptoms of PD and the emerging disability in order to provide better care and delay functional limitations. This current study investigated the disability of moderate and advanced PwP at variable cognitive and walking status, which were assessed by WHODAS 2.0 with the objective of identify the associations between ambulation, cognitive status, and disability.

## Methods

### Participants and Data Collection

**Figure 1:**
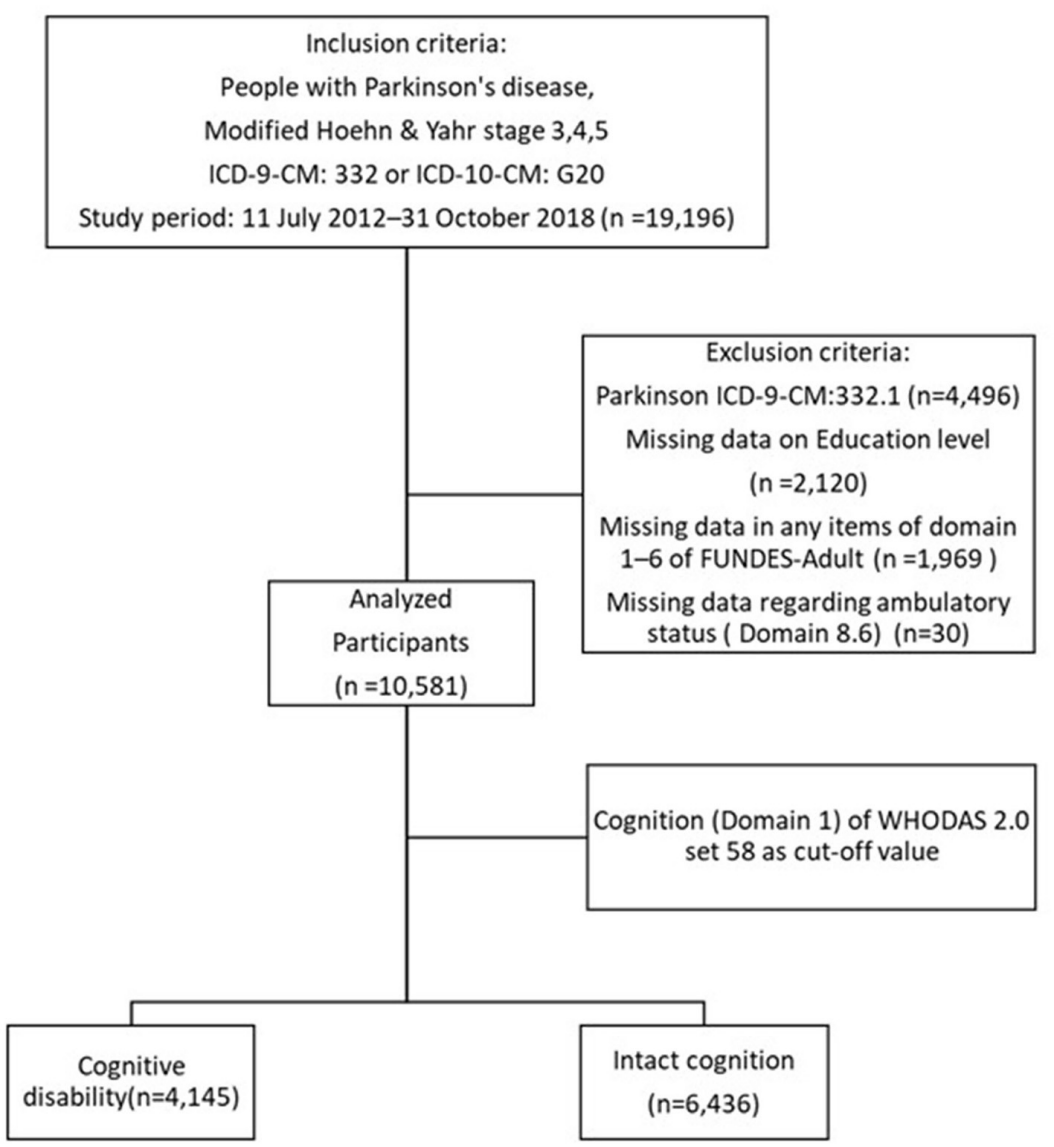
The study participants selection flow chart.

The data from a total of 19,196 moderate and advanced PwP (Hoehn and Yahr, H&Y, stage 3–5) were collected between July 2012 and October 2018 from a registry of disability evaluation and functional assessment established by the Taiwan Data Bank of Persons with Disability. The database was established by the Ministry of Health and Welfare in Taiwan which stipulates that only PwP with modified H&Y stages 3, 4, and 5 are eligible to receive disability certification and corresponding benefits. These may include all persons who are eligible for the first time (first time reaching the disease statue of H&Y stages 3–5) and those who are extending their disability certification. Applicants with PD were selected from the database via the International Classification of Diseases (ICD), Ninth Revision, Clinical Modification (ICD-9-CM) and ICD tenth Revision, Clinical Modification (ICD-10-CM) diagnosis codes ICD-9-CM 332 and ICD-10-CM G20. After excluding those with secondary parkinsonism (ICD-9-CM 332.1), omitted or missing data regarding the patient's ambulatory status, WHODAS 2.0 domains, and those who refused to answer, 10,581 subjects were analyzed (Figure 1).

The FUNDES-Adult was administered by multiple certified personnel from different hospitals, including social workers, physical, and occupational therapists from July 2012 and October 2018. For each study participant, basic demographic data including age, gender, residence, employment status, education level, family economic status, urbanization level, modified H&Y stage were collected. Following this, the six WHODAS 2.0 domains of cognition (domain 1), mobility (domain 2), self-care (domain 3), getting along with others (domain 4), household activities component of the life activities domain (domain 5-1, household activities), and participation in society (domain 6) were obtained by asking the participants to rate the extent to which their disabilities interfered with their lives in the preceding 30 days. Each domain consisted of 4–8 questions and a 5-point scale was used to assess the level of difficulty in the activities in each domain (0 = no difficulty, 1 = mild difficulty, 2 = moderate difficulty, 3 = severe difficulty, 4 = extreme difficulty). For example, domain 1 (cognition) consists of assessments of the following items: concentrating on something for 10 min, remembering to do important things, analyzing, and finding solutions to problems in day-to-day life, learning a new task, understanding what people say and starting and maintaining a conversation. For each item in the cognition domain, a score ranging from 0 to 4 is assigned. The total score is the sum of all the items, which ranges from 0 (best performance) to 20 (worst performance). The absolute score would be transformed to the standardized score for each domain were calculated based on the manual for WHODAS and ranged from 0 to 100, with higher scores indicating greater difficulty. The standardized scores were then summated to form the total score. The questionnaire was administered to the participants or the participants' caregivers if the participants could not answer the questions themselves. Domain 8.6 of the FUNDES-Adult was obtained to determine the patient's walking status, which is assessed by asking the applicant to walk in a straight line for 3 m and then return to the initial location on the spot in front of the interviewer. This aspect of motor capacity was judged with or without assistive technology and personal assistance. Walking statuses were defined as ambulatory/assisted ambulatory (domain 8.6 score from 0 through 3) and non-ambulatory (score of 4) if extreme difficulty is encountered and total assistance is need in an attempt to walk.

### Statistical Analysis

**Figure 2:**
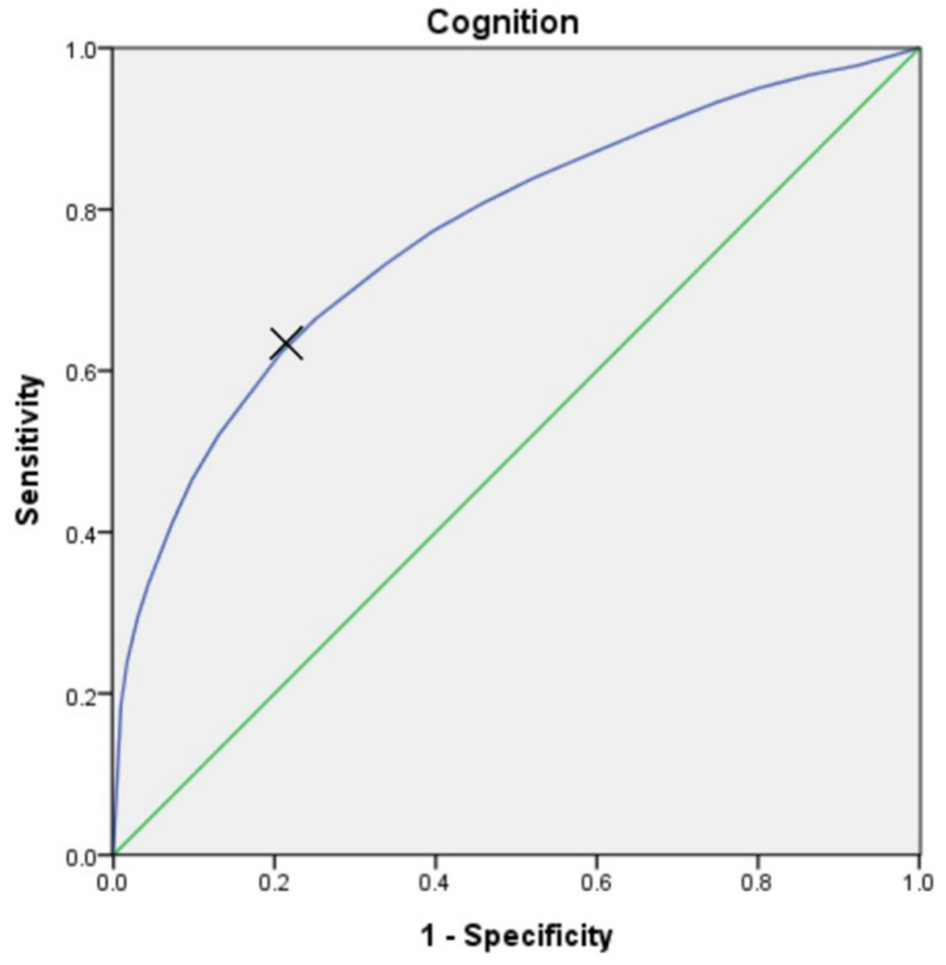
The Receiver Operating Characteristic (ROC) curve, which was utilized to predict the cognitive disability (score of WHODAS 2.0 domain 1) in PwP by ambulatory status. At the cut-off value of 58, the area under the curve was 0.77, with sensitivity and specificity of 62% and 79%, respectively.

The optimal cut-off point on the Receiver Operating Characteristic (ROC) curve was determined using the Youden's index for the highest sensitivity and specificity in predicting cognitive disability in PwP by the ambulatory status based on their score of cognition (WHODAS 2.0 domain 1). The cut-off value was 58 with area under curve was 0.77, with sensitivity and specific of 62 and 79%, respectively (Figure 2). Demographic characteristics of age and gender were employed via the χ^2^ test and one-way ANOVA test were used for comparisons between groups. Non-parametric regression was applied to compare the impact of cognition and ambulation on different WHODAS 2.0 domains. SAS (version 9.2, SAS institute, Inc., Cary, NC, USA) was utilized to perform the analyses and statistical significance was set at *p* < 0.05.

## Results

Of the analyzed 10,581 PwP, 4,145 were categorized as having cognitive disability and the other 6,436 were considered cognitively intact using the cut-off value at 58 score of the cognition domain (domain 1) in WHODAS 2.0. The mean age was 76.4 ± 8.3 years old in the PwP with cognition disability, compared to 71.1 ± 9.9 years old in the cognition intact counterpart. Demographic data of these two groups revealed that the gender distribution was similar; however, significant differences were noted for age, education level, work status residence, severity of disability and modified H&Y stage. All domains of the WHODAS 2.0 were significantly higher in those with cognitive disability than those without, representing higher disability levels in all aspects of daily living (Table 1).

**Table 1:** Demographic data of all the study participants.

	**Cognitive disability (*n* = 4,145)**	**Intact cognition (*n* = 6,436)**	***p*-value**
Female	2,104 (50.8)	3,205(49.8)	0.33
Age (y/o)	76.4 ± 8.3	71.1 ± 9.9	<0.001
18–64	411 (9.9)	1,690 (26.3)	
Education			<0.001
College or higher	92 (2.2)	131 (2.0)	
Senior high school	305 (7.4)	552 (8.6)	
Junior high school	378 (9.1)	1,125 (17.5)	
Primary school	2,550 (61.5)	4,033 (62.7)	
No education	820 (19.8)	595 (9.3)	
Residence			<0.001
Community dwelling	3,336 (80.5)	6,044 (93.9)	
Institution	809 (19.5)	392 (6.1)	
Urbanization level			<0.001
Rural	567 (13.7)	724 (11.3)	
Suburban	1,505 (36.3)	2,219 (34.5)	
Urban	2,073 (50.0)	3,493 (54.3)	
Work status			<0.001
Employment	13 (0.3)	219 (3.4)	
Unemployment	4,132 (99.7)	6,217 (96.6)	
Family economic status			0.51
General	4,112 (99.2)	6,392 (99.3)	
Middle low and low	33 (0.8)	44 (0.7)	
Modified H&Y Stage			<0.001
Stage 3	879 (21.2)	3,052 (47.4)	
Stage 4	1,658 (40.0)	2,737 (42.5)	
Stage 5	1,608 (38.8)	647 (10.1)	
WHODAS 2.0			
Cognition (domain 1)	81.1 ± 14.2	29.0 ± 17.1	<0.001
Mobility (domain 2)	75.3 ± 22.8	47.8 ± 24.9	<0.001
Self-care (domain 3)	52.6 ± 34.1	30.7 ± 25.2	<0.001
Getting along (domain 4)	83.8 ± 19.7	44.2 ± 27.0	<0.001
Life activities (domain 5-1)	85.2 ± 32.7	60.6 ± 37.9	<0.001
Participation in society (domain 6)	62.1 ± 23.1	40.4 ± 21.1	<0.001
Summary score	72.8 ± 15.2	40.8 ± 16.3	<0.001

*PwP, People with Parkinson's disease; H&Y, Modified Hoehn-Yahr Stage; WHODAS, WHO Disability Assessment Scale. Data was presented as number (percentage) or mean ± standard deviation*.

Further taking walking status into the analysis for the impact on disability, the overall PwP were grouped according to the combination of two conditions: intact cognition/cognitive disability and ambulatory/non-ambulatory status (Table 2). There were 1,231 patients (11.6%) categorized into the cognitive disability/ambulatory or assisted ambulatory group, 4,673 (44.2%) ambulatory or assisted ambulatory/intact cognition, 2,914 (27.5%) in the cognitive disability/non-ambulatory group and 1,763 (16.7%) in the intact cognition/non-ambulatory group. There were statistically significant age differences within the four groups, with non-ambulatory and cognition disability PwP being the oldest (77.6 ± 7.7 years old) and the ambulatory and intact cognition group being the youngest (69.7 ± 10.1 years old, *p* for trend). We found that the presence of cognitive disability is associated with higher individual domain and total scores in both ambulatory and non-ambulatory groups (*p* < 0.05). Non-ambulatory PwP with cognitive disability had the highest level of disability (76.5 ± 14.4), followed by ambulatory PwP with cognitive disability (64.2 ± 13.4), non-ambulatory PwP with intact cognition (50.6 ± 15.4) and ambulatory PwP with intact cognition (37.2 ± 15.1). The same trend holds true for each individual domain of the WHODAS 2.0.

**Table 2:** Subgrouping of all study participants based on ambulation and cognition, *n* = 10,581.

	**Ambulatory**		**Non-ambulatory**		***p* for trend**
	**Cognitive disability (*n* = 1,231)**	**Intact cognition (*n* = 4,673)**	**Cognitive disability (*n* = 2,914)**	**Intact cognition (*n* = 1,763)**	
Female	594 (48.3)	2,272 (48.6)	1,510 (51.8)	933 (52.9)	0.46
Age (y/o)	73.6 ± 8.8	69.7 ± 10.1	77.6 ± 7.7	75.0 ± 8.3	<0.001
H&Y Stage					<0.001
Stage 3	533 (43.3)	2,677 (57.3)	346 (11.9)	375 (21.3)	
Stage 4	587 (47.7)	1,815 (38.8)	1,071 (36.8)	922 (52.3)	
Stage 5	111 (9.0)	181 (3.9)	1,497 (51.4)	466 (26.4)	
WHODAS 2.0					
Cognition (domain 1)	73.6 ± 11.5	27.1 ± 17.0	84.2 ± 14.0	34.1 ± 16.4	<0.001
Mobility (domain 2)	61.4 ± 22.4	41.2 ± 21.7	81.2 ± 20.3	65.4 ± 24.1	<0.001
Self-care (domain 3)	42.1 ± 28.9	27.3 ± 22.3	57.1 ± 35.2	39.6 ± 29.9	<0.001
Getting along (domain 4)	74.7 ± 22.1	40.2 ± 25.6	87.7 ± 17.2	54.9 ± 27.8	<0.001
Life activities (domain 5-1)	80.9 ± 33.9	55.7 ± 36.1	87.0 ± 32.0	73.6 ± 39.5	<0.001
Participation in society (domain 6)	55.4 ± 21.7	37.8 ± 20.3	64.9 ± 23.0	47.2 ± 21.6	<0.001

*WHODAS, WHO Disability Assessment Scale; H&Y, Hoehn and Yahr Stage. Data was presented as number (percentage) or mean ± standard deviation*.

In order to further delineate the isolated association between ambulatory status and cognition with the functional disability, a non-parametric regression was conducted. Age, education, and urbanization level, location of residence (whether they are community dwelling or not), modified H&Y were also included in the regression in order to examine their effects (Table 3). The results showed that non-ambulatory and cognitive disability were significantly associated with the severity of disability in every aspect. The effect size (β) of cognitive disability was consistently greater than that of ambulatory status in four domains of self-care (14.7 ± 0.7 vs. 10.6 ± 0.7), getting along with others (35 ± 0.5 vs. 12 ± 0.6), life activities (18.1 ± 0.8 vs. 11.3 ± 0.8), and participation (22 ± 0.6 vs. 11.3 ± 0.6). Similarly, disease stage (modified H&Y stage) also showed a statistically significant effect in all four domains, though the effect sizes were still smaller compared to either that of cognition and ambulation status. Institutional residents, age, and level of urbanization also appears to have impact on some domains whereas education level does not.

**Table 3:** Non-parametric regression comparing the effects of cognition and ambulation on WHODAS domains 3–6.

	**Self-care (domain 3)**			**Getting along (domain 4)**			**Life activities (domain 5-1)**			**Participation in society (domain 6)**		
	**β**	**SE**	***P*-value**	**β**	**SE**	***P*-value**	**β**	**SE**	***P*-value**	**β**	**SE**	***P*-value**
Cognitive disability (ref = Intact cognition)	14.7	0.7	<0.001	35	0.5	<0.001	18.1	0.8	<0.001	22	0.6	<0.001
Non-ambulatory (ref = Ambulatory)	10.6	0.7	<0.001	12	0.6	<0.001	11.3	0.8	<0.001	11.3	0.6	<0.001
Age ≥ 65 (ref = 18–64)	−2.0	0.9	0.02	1.1	0.7	0.12	5.2	1.0	<0.001	−7.8	0.8	<0.001
Institution Residence (ref = Community dwelling)	3.3	1.0	<0.001	2.0	0.8	0.01	2.8	1.1	0.01	0.7	0.9	0.40
Urbanization level												
Suburban (ref = urban)	0.6	0.6	0.36	−0.5	0.5	0.32	−1.0	0.7	0.15	0.2	0.6	0.77
Rural (ref = urban)	−3.0	0.9	0.002	−1.3	0.7	0.07	−2.2	1.1	0.03	−1.7	0.8	0.04
Education ≤ 6 years (ref ≥ 6 years)	1.6	0.8	0.06	−0.7	0.6	0.26	0.8	0.9	0.36	−1.1	0.7	0.12
Modified H&Y Stage												
Stage 4 (ref = Stage 3)	2.0	0.7	0.003	3.2	0.5	<0.001	5.8	0.8	<0.001	3.4	0.6	<0.001
Stage 5 (ref = Stage 3)	5.0	1.0	<0.001	9.2	0.8	<0.001	5.5	1.1	<0.001	3.5	0.8	<0.001

## Discussion

The findings from the present study revealed that non-ambulatory status and cognitive disability were both independently associated with the severity of disability, and the contribution of cognition was greater than ambulation. Unlike the PD-specific *Unified Parkinson's Disease Rating Scale* (UPDRS) (22), the present study utilized WHODAs 2.0 to evaluate the multiple aspects of disability, which can be assessed by qualified medical personnel who do not necessarily have to be physicians, is less time-consuming and enables the evaluation of disability under the ICF framework. Using WHODAS 2.0, the present study showed that the disability in moderate to advanced PwP is not merely limited to motor-related life activities, but affects one's ability to understand and communicate, getting along with people and participation in society and such difficulties become more prominent as cognition levels deteriorate and as independent or assisted ambulation is lost. Out of the four study groups, non-ambulatory PwP with cognitive disability have the most severe disabilities in all domains of daily functioning. These findings are consistent with previous findings, indicating that functional decline in PD results from motor impairment and is likely aggravated by concomitant cognitive impairment (23–25). Furthermore, based on the results of our non-parametric analysis, we hypothesize that as a determinant of functional disability, cognition is likely to be more important than ambulation status in later stages of PD.

It has been demonstrated that axial impairment (postural instability and gait difficulty) is strongly associated with the disability, functional dependence, and poor quality of life in non-demented mild to moderate PwP whereas there is relatively little impact of cognitive performance on functional outcome. (26) The loss of functional dependency tends to occur at the transition between H&Y stages 2–3 which characterizes the emergence of postural instability and possibly loss of independent ambulation (27, 28). It is thought that gait impairment portends the loss of many gait-dependent activities and thus, the effect of early manifesting gait disturbance such as freezing and postural instability can become especially prominent. The cognition is more likely to be spared or mild impairment when the affected persons are still in their early stages of PD. In contrast to the findings in early disease, we observed that ambulatory PwP with cognitive disability had higher overall disability compared to those who were cognitively intact but non-ambulatory, suggesting that cognitive function contributes more to disability than mobility and ambulation status. This illustrates the important concept of disparity between mobility and “functional mobility” in PD (29): despite that the ambulatory/cognition disability group had preserved physiological ability of moving independently or with assistance, they still experienced more difficulties in performing life activities and in maintaining an active and social life.

The findings of the present study may indicate the necessity of cognitive interventions, such medical therapy or cognitive training to prevent the disability of PwP. So far, there is evidence to support the efficacy and safety of acetylcholinesterase inhibitors such as rivastigmine with regarding slowing the cognitive impairment in PDD (30, 31). It will be worthwhile to investigate whether this benefit can translate into improvements in functional ability. Non-pharmacological options, on the other hand, may be preferable in those who are already burdened by polypharmacy or in cases who are already suffering from drug side effects (32). Compelling evidence from randomized controlled trials have found that cognitive training can improve cognitive domains known to be impaired in PD, such as working memory, processing speed, and executive function (33, 34). Cognitive training can also reduce motor complications such as freezing of gait (35, 36). Another study found that cognitive rehabilitation of a 3-month duration can lead to improved cognition and reduced functional disability measured by WHODAS. Moreover, these improvements were sustained after a period of 18 months (37). The targeted effects of aerobic exercise on motor and cognitive circuitry is also promising, with observed reduction in motor symptom severity and non-motor symptoms of fatigue, depression, and executive function (38). Since the aforementioned studies included mild to moderate PD or exclude patients with dementia, whether the same benefits can be replicated in more severely affected patients should be further investigated. The impact magnitude of physical exercise on cognitive function and real-world activities needs to be further clarified.

As a cross-sectional study, the limitations of this study include its sampling population, which consists of moderate to advanced PwP, restricting the applicability of the aforementioned findings to people with early stage or mild disease and precludes inferences about causality. Secondly, due to the retrospective nature of the study, the group with cognition disability was defined using the more generic cognition domain score on the FUNDES-Adult instead of tests such as the Mini Mental State exam or the Montreal Cognitive Assessment and definitions of the Movement Disorder Society criteria for PDD (39); cognition while those in this group may not strictly fulfill the diagnosis of “dementia” and may even include those with non-demented cognitive impairment, this method may have wider applicability in the primary care setting, providing an easier assessment of cognitive status for Physical Medicine and Rehabilitation physicians, physical therapists, or clinicians less familiar PDD diagnosis or when formal comprehensive neuropsychological testing cannot be conducted. Similarly, ambulation and walking status was evaluated by asking the patient to walk back and fro 3 m (domain 8.6 of FUNDES-Adult) instead of tests such as the Timed up and Go test. The 3-m walking course has been found to be a valid form of assessment of walking status compared to longer course lengths and is employed on a national level by the Taiwanese government after being tested for its validity and reliability (5, 8, 40). Thirdly, we cannot gather information on disease duration, treatment status, comorbidities in our cohort, for instance, co-existing cerebrovascular disease, amyloid pathology, and mood disorders, the impact of which can potentially confound our analysis but due to the advantage of having a large sample size, the evidence nonetheless still supports our hypothesis. Finally, the ambulation assessment by to and fro 3-m walk is episodic and the PwP may in their off status, which may result in miss categorization.

## Conclusion

In mid-to-late stages of disease, PwP may experience significant functional disability resulting from dependent ambulation and cognitive impairment. This study identified that cognitive status has a greater impact on functional disability compared to ambulation status in those who are modified H-Y stages 3 and above. Future research must determine whether methods for secondary prevention of cognitive decline in this population can delay or mitigate functional dependency and reduce the social and economic burden brought about by this neurodegenerative disease.

## Data Availability Statement

The datasets generated for this study are available on request to the corresponding author.

## Ethics Statement

The studies involving human participants were reviewed and approved by the Joint Institutional Review Board of Taipei Medical University (N201805048). Written informed consent for participation was not required for this study in accordance with the national legislation and the institutional requirements.

## Author Contributions

CW: data analysis and manuscript writing. LC: study design and manuscript revision. DW: data collection. W-CC, C-FY, and H-FL: study design and data collection. CH: study design, data analysis, and manuscript revision. T-HL: study design, data collection, data analysis, and manuscript revision.

### Conflict of Interest

The authors declare that the research was conducted in the absence of any commercial or financial relationships that could be construed as a potential conflict of interest.

## References

[1] Chaudhuri KR Healy DG Schapira AH. Non-motor symptoms of Parkinson's disease: diagnosis and management. Lancet Neurol. (2006) 5:235–45. 10.1016/S1474-4422(06)70373-8 16488379

[2] Samii A Nutt JG Ransom BR. Parkinson's disease. Lancet. (2004) 363:1783–93. 10.1016/S0140-6736(04)16305-8 15172778

[3] Global Burden of Disease 2016 Neurology Feigin VL Nichols E Alam T Bannick MS Beghi E Global, regional, and national burden of neurological disorders, 1990-2016: a systematic analysis for the Global Burden of Disease Study 2016. Lancet Neurol. (2019) 18:459–80. 10.1016/S1474-4422(18)30499-X 30879893 PMC6459001

[4] Leonardi M Bickenbach J Ustun TB Kostanjsek N Chatterji S MHADIE Consortium. The definition of disability: what is in a name? Lancet. (2006) 368:1219–21. 10.1016/S0140-6736(06)69498-1 17027711

[5] Yen CF Hwang AW Liou TH Chiu TY Hsu HY Chi WC. Validity and reliability of the functioning disability evaluation scale-adult version based on the WHODAS 2.0–36 items. J Formos Med Assoc. (2014) 113:839–49. 10.1016/j.jfma.2014.08.008 25294100

[6] Chiu TY Yen CF Chou CH Lin JD Hwang AW Liao HF. Development of traditional Chinese version of World Health Organization disability assessment schedule 2.0 36–item (WHODAS 2.0) in Taiwan: validity and reliability analyses. Res Dev Disabil. (2014) 35:2812–20. 10.1016/j.ridd.2014.07.009 25094056

[7] Federici S Bracalenti M Meloni F Luciano JV. World Health Organization disability assessment schedule 2.0: an International systematic review. Disabil Rehabil. (2017) 39:2347–80. 10.1080/09638288.2016.1223177 27820966

[8] Chiu WT Yen CF Teng SW Liao HF Chang KH Chi WC. Implementing disability evaluation and welfare services based on the framework of the International classification of functioning, disability and health: experiences in Taiwan. BMC Health Serv Res. (2013) 13:416. 10.1186/1472-6963-13-416 24125482 PMC3853212

[9] Teng SW Yen CF Liao HF Chang KH Chi WC Wang YH. Evolution of system for disability assessment based on the International classification of functioning, disability, and health: a Taiwanese study. J Formos Med Assoc. (2013) 112:691–8. 10.1016/j.jfma.2013.09.007 24099681

[10] Post B Merkus MP de Haan RJ Speelman JD. Prognostic factors for the progression of Parkinson's disease: a systematic review. Mov Disord. (2007) 22:1839–51. 10.1002/mds.21537 17595026

[11] Schrag AY Ben-Shlomo Quinn N. How common are complications of Parkinson's disease? J Neurol. (2002) 249:419–23. 10.1007/s004150200032 11967646

[12] Weintraub D Moberg PJ Duda JE Katz IR Stern MB. Effect of psychiatric and other nonmotor symptoms on disability in Parkinson's disease. J Am Geriatr Soc. (2004) 52:784–8. 10.1111/j.1532-5415.2004.52219.x 15086662

[13] Emre M. Dementia associated with Parkinson's disease. Lancet Neurol. (2003) 2:229–37. 10.1016/S1474-4422(03)00351-X 12849211

[14] Aarsland D Kurz MW. The epidemiology of dementia associated with Parkinson disease. J Neurol Sci. (2010) 289:18–22. 10.1016/j.jns.2009.08.034 19733364

[15] Emre M Aarsland D Brown R Burn DJ Duyckaerts C Mizuno Y. Clinical diagnostic criteria for dementia associated with Parkinson's disease. Mov Disord. (2007) 22:1689–707. 10.1002/mds.21507 17542011

[16] Hely MA Morris JG Reid WG Trafficante R. Sydney multicenter study of Parkinson's disease: non-L-dopa-responsive problems dominate at 15 years. Mov Disord. (2005) 20:190–9. 10.1002/mds.20324 15551331

[17] Goldman JG Vernaleo BA Camicioli R Dahodwala N Dobkin RD Ellis T. Cognitive impairment in Parkinson's disease: a report from a multidisciplinary symposium on unmet needs and future directions to maintain cognitive health. NPJ Parkinsons Dis. (2018) 4:19. 10.1038/s41531-018-0055-3 29951580 PMC6018742

[18] Papapetropoulos S Ellul J Polychronopoulos P Chroni E. A registry-based, case-control investigation of Parkinson's disease with and without cognitive impairment. Eur J Neurol. (2004) 11:347–51. 10.1111/j.1468-1331.2004.00826.x 15142230

[19] Varalta V Picelli A Fonte C Amato S Melotti C Zatezalo V. Relationship between cognitive performance and motor dysfunction in patients with Parkinson's disease: a pilot cross-sectional study. Biomed Res Int. (2015) 2015:365959. 10.1155/2015/365959 25918713 PMC4396143

[20] Burn DJ Rowan EN Allan LM Molloy S O'Brien JT McKeith IG. Motor subtype and cognitive decline in Parkinson's disease, Parkinson's disease with dementia, and dementia with Lewy bodies. J Neurol Neurosurg Psychiatry. (2006) 77:585–9. 10.1136/jnnp.2005.081711 16614017 PMC2117449

[21] Plotnik M Dagan Y Gurevich T Giladi N Hausdorff JM. Effects of cognitive function on gait and dual tasking abilities in patients with Parkinson's disease suffering from motor response fluctuations. Exp Brain Res. (2011) 208:169–79. 10.1007/s00221-010-2469-y 21063692

[22] Goetz CG Tilley BC Shaftman SR Stebbins GT Fahn S Martinez-Martin P. Movement disorder society-sponsored revision of the Unified Parkinson's Disease Rating Scale (MDS-UPDRS): scale presentation and clinimetric testing results. Mov Disord. (2008) 23:2129–70. 10.1002/mds.22340 19025984

[23] Stella F Banzato CEM Quagliato EMAB Viana MA Christofoletti G. Dementia and functional decline in patients with Parkinson's disease. Dement Neuropsychol. (2008) 2:96–101. 10.1590/S1980-57642009DN20200004 29213550 PMC5619577

[24] Lee HH Hong CT Wu D Chi WC Yen CF Liao HF. Association between ambulatory status and functional disability in elderly people with Dementia. Int J Environ Res Public Health. (2019) 16:2168. 10.3390/ijerph16122168 31248158 PMC6616473

[25] Chen JH Hong CT Wu D Chi WC Yen CF Liao HF. Dementia-related functional disability in moderate to advanced Parkinson's disease: assessment using the World Health Organization disability assessment schedule 2.0. Int J Environ Res Public Health. (2019) 16:E2230. 10.3390/ijerph16122230 31238603 PMC6617247

[26] Muslimovic D Post B Speelman JD Schmand B de Haan RJ. Determinants of disability and quality of life in mild to moderate Parkinson disease. Neurology. (2008) 70:2241–7. 10.1212/01.wnl.0000313835.33830.80 18519873

[27] Shulman LM Gruber-Baldini AL Anderson KE Vaughan CG Reich SG Fishman PS. The evolution of disability in Parkinson disease. Mov Disord. (2008) 23:790–6. 10.1002/mds.21879 18361474

[28] Hoehn MM Yahr MD. Parkinsonism: onset, progression and mortality. Neurology. (1967) 17:427–42. 10.1212/WNL.17.5.427 6067254

[29] Bouça-Machado R Maetzler W Ferreira JJ. What is functional mobility applied to Parkinson's disease? J Parkinsons Dis. (2018) 8:121–30. 10.3233/JPD-171233 29480225 PMC5836402

[30] Wang HF Yu JT Tang SW Jiang T Tan CC Meng XF. Efficacy and safety of cholinesterase inhibitors and memantine in cognitive impairment in Parkinson's disease, Parkinson's disease dementia, and dementia with Lewy bodies: systematic review with meta-analysis and trial sequential analysis. J Neurol Neurosurg Psychiatry. (2015) 86:135–43. 10.1136/jnnp-2014-307659 24828899

[31] Seppi K Ray Chaudhuri K Coelho M Fox SH Katzenschlager R Perez Lloret S. Update on treatments for nonmotor symptoms of Parkinson's disease-an evidence-based medicine review. Mov Disord. (2019) 34:180–98. 10.1002/mds.27602 30653247 PMC6916382

[32] Antonini A Moro E Godeiro C Reichmann H. Medical and surgical management of advanced Parkinson's disease. Mov Disord. (2018) 33:900–8. 10.1002/mds.27340 29570862

[33] Leung IH Walton CC Hallock H Lewis SJ Valenzuela M Lampit A. Cognitive training in Parkinson disease: a systematic review and meta-analysis. Neurology. (2015) 85:1843–51. 10.1212/WNL.0000000000002145 26519540 PMC4662707

[34] Edwards JD Hauser RA O'Connor ML Valdés EG Zesiewicz TA Uc EY. Randomized trial of cognitive speed of processing training in Parkinson disease. Neurology. (2013) 81:1284–90. 10.1212/WNL.0b013e3182a823ba 24014503 PMC3806923

[35] Walton CC Mowszowski L Gilat M Hall JM O'Callaghan C Muller AJ. Cognitive training for freezing of gait in Parkinson's disease: a randomized controlled trial. NPJ Parkinsons Dis. (2018) 4:15. 10.1038/s41531-018-0052-6 29796409 PMC5959878

[36] Mirelman A Bonato P Camicioli R Ellis TD Giladi N Hamilton JL. Gait impairments in Parkinson's disease. Lancet Neurol. (2019) 18:697–708. 10.1016/S1474-4422(19)30044-4 30975519

[37] Díez-Cirarda M Ojeda N Peña J Cabrera-Zubizarreta A Lucas-Jiménez O Gómez-Esteban JC. Long-term effects of cognitive rehabilitation on brain, functional outcome and cognition in Parkinson's disease. Eur J Neurol. (2018) 25:5–12. 10.1111/ene.13472 28940855 PMC5765471

[38] Petzinger GM Fisher BE McEwen S Beeler JA Walsh JP Jakowec MW. Exercise-enhanced neuroplasticity targeting motor and cognitive circuitry in Parkinson's disease. Lancet Neurol. (2013) 12:716–26. 10.1016/S1474-4422(13)70123-6 23769598 PMC3690528

[39] Kiesmann M Chanson JB Godet J Vogel T Schweiger L Chayer S. The movement disorders society criteria for the diagnosis of Parkinson's disease dementia: their usefulness and limitations in elderly patients. J Neurol. (2013) 260:2569–79. 10.1007/s00415-013-7018-8 23835635

[40] Lyons JG Heeren T Stuver SO Fredman L. Assessing the agreement between 3-meter and 6-meter walk tests in 136 community-dwelling older adults. J Aging Health. (2015) 27:594–605. 10.1177/0898264314556987 25376604 PMC4522919

